# Preventing MQTT Vulnerabilities Using IoT-Enabled Intrusion Detection System

**DOI:** 10.3390/s22020567

**Published:** 2022-01-12

**Authors:** Muhammad Husnain, Khizar Hayat, Enrico Cambiaso, Ubaid U. Fayyaz, Maurizio Mongelli, Habiba Akram, Syed Ghazanfar Abbas, Ghalib A. Shah

**Affiliations:** 1 Al-Khwarizmi Institute of Computer Science (KICS), University of Engineering and Technology (UET), Lahore 39161, Pakistan; muhammad.husnain@kics.edu.pk (M.H.); khizar.hayat@kics.edu.pk (K.H.); ubaid.fayyaz@kics.edu.pk (U.U.F.); habiba.akram@kics.edu.pk (H.A.); ghazanfar.abbas@kics.edu.pk (S.G.A.); ghalib@kics.edu.pk (G.A.S.); 2 Consiglio Nazionale delle Ricerche (CNR), IEIIT Institute, 16149 Genoa, Italy; maurizio.mongelli@cnr.it

**Keywords:** Internet of Things, intrusion detection system, MQTT protocol, network firewall, network attacks, IoT vulnerabilities

## Abstract

The advancement in the domain of IoT accelerated the development of new communication technologies such as the Message Queuing Telemetry Transport (MQTT) protocol. Although MQTT servers/brokers are considered the main component of all MQTT-based IoT applications, their openness makes them vulnerable to potential cyber-attacks such as DoS, DDoS, or buffer overflow. As a result of this, an efficient intrusion detection system for MQTT-based applications is still a missing piece of the IoT security context. Unfortunately, existing IDSs do not provide IoT communication protocol support such as MQTT or CoAP to validate crafted or malformed packets for protecting the protocol implementation vulnerabilities of IoT devices. In this paper, we have designed and developed an MQTT parsing engine that can be integrated with network-based IDS as an initial layer for extensive checking against IoT protocol vulnerabilities and improper usage through a rigorous validation of packet fields during the packet-parsing stage. In addition, we evaluate the performance of the proposed solution across different reported vulnerabilities. The experimental results demonstrate the effectiveness of the proposed solution for detecting and preventing the exploitation of vulnerabilities on IoT protocols.

## 1. Introduction

The Internet of Things (IoT) is a collection of smart objects connected to the Internet to provide different services for the ease of human beings [1]. IoT introduced many innovative concepts such as smart home, smart city, smart traffic system, smart parking, smart industry, and smart wearable, all of which enhance human life. Although IoT contributes many novel applications in our daily life, one of its main concerns refers to IoT devices security. Recent cyber-security incidents exposed the security pitfalls and vulnerabilities in IoT devices [2,3,4]. Once an IoT device is connected to the Internet, it becomes an attractive target for attackers seeking to access the device content and control for malicious activities [5].

Generally, IoT devices are equipped with limited storage and processing power [6]. IoT device manufacturers mostly focus on adding new attractive features/functionalities in IoT devices, simplifying the design to make the devices smarter and more cost-effective rather than making them secure [7]. Manufacturers are well aware of security hazards, but they either ignore it or treat it as an afterthought feature due to the race to market [8]. Thus, the end product comes with a wide set of attractive features but with inherited security pitfalls, hence becoming an attractive target for cyber-criminals.

The resource constraint feature of IoT devices makes them vulnerable to different attacks [9] such as spoofing attacks, denial of service (DoS) attacks, replay attacks, etc. In a recent work, it was reported that more than 25% of the compromised devices in a botnet consist of smart home IoT devices such as smart TV, smart cameras, etc. [10]. The famous Mirai attack gained control over thousands of IoT devices by exploiting the default credentials and launched a distributed DoS (DDoS) attack on critical servers [11]. Hence, there is an immense need to protect IoT device vulnerabilities. By design, IoT devices are not properly secured to counter vulnerability attacks [6]. A vulnerability is a loophole in a system that provides a fertile ground for cyber-attacks. Standing to the Internet standards body, i.e., Internet Engineering Task Force (IETF), a vulnerability (https://datatracker.ietf.org/doc/html/rfc4949, accessed on 12 December 2021) is “a flaw or weakness in a system’s design, implementation, or operation and management that could be exploited to violate the system’s security policy”.

IoT vulnerabilities are mostly due to insufficient product testing, race to market, and lack of proper legislation [7,12]. Not long ago, in a study, HP claimed that 70% of IoT devices have vulnerabilities (https://www8.hp.com/us/en/hp-news/press-release.html?id=1744676, accessed on 12 December 2021). Likewise, in the most recent report of the World Economic Forum (WEF) (https://www3.weforum.org/docs/WEF_Global_Risks_Report_2019.pdf, accessed on 12 December 2021), it is revealed that the cyber-attacks are ranked among the top 10 global risks in terms of impact.

Over the past few years, a number of vulnerabilities have been revealed in IoT devices. Recently, Zhen Ling [13] identified vulnerabilities in the communication protocol of a smart plug (Edimax SP-2101W)—an extensively installed IoT device for home automation—that could enable attacks such as brute force, scanning, spoofing, and firmware attacks. Similarly, another study revealed that thousands of consumer IoT devices exposed over the Internet are potentially vulnerable, and most of the devices had vulnerabilities due to the outdated protocol versions [14]. Likewise, the authors in [15] identified that most of the protocol exploits occur due to extraneous features in a protocol that are never used by an underlying IoT device/application. The protocol implementation vulnerabilities may cause devastating cyber-attacks on billions of IoT devices and IoT-driven production systems that can result in information disclosure, ransomware installation [15], etc. According to a recent study [16], it was disclosed that on average, an insecure IoT device contains 25 vulnerabilities. Furthermore, 60% of the IoT devices contain vulnerable interfaces and firmware, while 70% do not use any encryption technique for communications [16]. Hence, there is an immense need to protect the vulnerabilities of IoT devices, especially those using unencrypted protocols for communication.

Most of the IoT devices are vulnerable due to bugs on protocols implementation, device management issues, or improper handling of communication messages [17]. In order to fix such issues, the developers need to patch the IoT devices through firmware updates. Unfortunately, most of the IoT devices are not capable of being updated as they are not designed to receive updates over-the-air [18]. Thus, it puts billions of IoT devices at risk, as they are incapable of receiving updates and hence remain unprotected, insecure, and vulnerable.

One of the methods to protect an IoT device from known vulnerabilities is the use of a network-based intrusion detection system (IDS). A network-based IDS can better protect the IoT device protocol vulnerabilities provided that it supports the protocols that IoT devices are using for communication. Moreover, it can protect the IoT devices from being exploited during the parsing stage by matching the rules to protect the IoT device vulnerabilities. As mentioned earlier, protocols such as MQTT or CoAP are frequently used by IoT devices. Unfortunately, the existing network-based IDSs do not support many of such protocols. Therefore, we propose a methodology for adding IoT protocols support in an open-source IDS, i.e., Suricata, in order to protect the vulnerabilities of the IoT devices, network, and protocols. The key focus of this work is to protect MQTT protocol vulnerabilities as it is the most used IoT protocol [19,20]. The major contributions of this work are as follows:

- We first analyze the recently reported MQTT protocol vulnerabilities and disclosed the major causes that are ignored while protocol implementation.
- Based on the analysis, we propose a robust parsing engine and integrated it with an existing open-source IDS in order to detect and prevent the IoT devices from MQTT protocol implementation vulnerabilities. To our best knowledge, it is the first time that protocol vulnerability attacks are prevented through a parsing engine at the IDS level before they reach the end device.
- Finally, in order to prove the effectiveness of the proposed solution, we deploy the proposed solution in a real-time network and tested it against multiple reported IoT protocol vulnerabilities.

The rest of the paper is structured as follows: Section 2 presents the literature review of a selection of recently exposed vulnerabilities in IoT devices along with existing solutions proposed for detecting and protecting the vulnerabilities. Section 3 investigates in detail the characteristics of known vulnerabilities targeting the MQTT protocol. Section 4 describes the proposed methodology for protecting the vulnerabilities in IoT devices, network, and protocols. Section 5 discusses the experimentation and demonstrates how the proposed methodology can safeguard the IoT vulnerabilities and stop both the inbound and outbound cyber-attacks. Finally, Section 6 concludes the paper and reports further work on the topic.

## 2. Literature Review

The rampant exploitation of vulnerabilities and security pitfalls in IoT devices are alarming the devastating effects of cyber-attacks in human life due to the widespread utilization of IoT devices in our daily life. Therefore, it is imperative to secure and protect the IoT devices from devastating cyber-attacks. Conventionally, two strategies are adopted to secure the IoT devices or networks from cyber-attacks. These include on-device security and network-based security [21]. The on-device security means the security shields that the manufacturer has included into the device by default. It includes the username, password, hash keys, or certificates to protect the IoT device from malicious activities. Vendors forge the on-device security mechanism with device firmware. On the other hand, the network-based security solutions mainly focus on securing the network from inbound attacks. Moreover, they also control the inbound and outbound communication of the devices. Despite the mature network defence technologies such as firewall, intrusion detection system, etc., the computer systems are exposed with more and more vulnerabilities [22]. Furthermore, the traditional network defence systems are inadequate for securing the IoT devices and network [11,21,23,24,25] due to lack of support for widely used IoT application layer protocols such as constrained application protocol (CoAP), message queuing telemetry transport (MQTT), etc. However, these vulnerabilities can be protected by adding the support of IoT protocols in existing security solutions. Therefore, the proactive discovery and protection of vulnerabilities of IoT devices have become imperative to safeguard the IoT network or device vulnerabilities before the attack.

Currently, firmware update is the most common approach to fix the discovered vulnerabilities in IoT devices. However, this feature is not available in every IoT device [26]. Only a few IoT vendors support the automatic update of firmware such as Atmel, Texas, etc. Moreover, the attackers may integrate some malware into the device firmware by exploiting the vulnerabilities in the firmware update technique. Therefore, firmware updates should also be secure from being exploited. The IoT devices retain poor support for patching or updating the firmware [27]. Once a vulnerability is reported, the vendor first verifies whether the reported vulnerability actually exists in their device or not. After the verification of the reported vulnerability, the vendor takes time to prepare the patch for the exploited vulnerability. Therefore, it takes a couple of months to patch a reported vulnerability, and until then, all the stakeholders are at the risk of facing the cyber-attacks. Hence, there is a need to provide such a system that is capable of protecting the vulnerable IoT devices until the vendor develops a patch or updates the device firmware.

According to the IETF RFC4949 (https://tools.ietf.org/html/rfc4949, accessed on 12 December 2021), system vulnerabilities can be categorized into three types: vulnerabilities in design, vulnerabilities in implementation, and vulnerabilities in operation and management. The design vulnerabilities occur due to issues in protocol design. These vulnerabilities are inherent in both implementation and operation. The implementation vulnerabilities occur due to the logical mistakes made by the developer while implementing the protocol specifications. The operation vulnerabilities occur due to some incident or event that alters the normal functionality of the system.

The rapid development of IoT devices and vendor’s race to market provoked the recklessness of security threats to a large extent [28]. The recent trends reported on the famous vulnerabilities reporting platforms, i.e., the National Vulnerability Database (NVD) (https://nvd.nist.gov, accessed on 12 December 2021) and Common Vulnerabilities and Exposures (CVE) (https://cve.mitre.org, accessed on 12 December 2021) databases, show that most of the IoT devices are vulnerable due to vulnerabilities in protocol design or vulnerabilities in protocol implementation. In a recent study [29], it is highlighted that the inadequate protocol implementation could be vulnerable to crafted or malformed packets and other types of attacks. The reason is that the inadequate implementation of protocol causes errors, buffer overflow, DoS attacks, etc., while handling the irregular crafted or malformed network traffic [29]. Likewise, in another study [14], the authors analyzed the vulnerabilities of three type of consumer IoT devices, i.e., smart TVs, printers, and webcams exposed over the Internet. The authors used a famous search engine, i.e., Shodan (https://www.shodan.io, accessed on 12 December 2021), to identify the consumer IoT devices and identified 156,680 consumer IoT devices exposed over the Internet. Afterwards, they scanned the identified IoT devices to assess the existence of potential vulnerabilities in identified devices by using a vulnerability assessment tool, i.e., Nessus. The study revealed that 12.92% of the identified consumer IoT devices had potential vulnerabilities, and most of them were due to outdated versions of communication protocols that can exploit the user information and privacy [14].

Hong et al. [15] identified that the attackers exploit extraneous features (i.e., features in a protocol that are never used by an underlying IoT device/application) to compromise the underlying IoT devices/applications. To address this issue, the authors proposed customizing a protocol by keeping the required protocol features and removing all the extraneous features from the protocol implementation. Similarly, Ling et al. [13] highlighted the severe vulnerabilities regarding the communication protocols and authentication mechanism in some popular smart home plugs. They exploited the communication protocols and gained authentication credentials by performing four type of attacks, i.e., device scanning attack, brute force attack, spoofing attack, and firmware attack. Finally, the authors gained access to the smart plug due to the insecure communication protocol and lack of device authentication. Likewise, in [30], the authors exploited the vulnerabilities of an IP camera and gained full control of IP camera by launching three attacks, which included a device-scanning attack, device-spoofing attack, and brute force attack. Their experiments revealed that an attacker can obtain the user passwords through the device-scanning attack with the probability of 98%.

The intelligent and connected vehicles such as Jeep or Tesla Model S were also found to be vulnerable to cyber-attacks aimed to get control of the vehicle in both standby and driving mode [7,31].

In another work (https://media.kasperskycontenthub.com/wp-content/uploads/sites/43/2018/12/13084354/ChargePoint-Home-security-research_final.pdf, accessed on 12 December 2021), the authors analyzed the hardware and software system of an electric vehicle charger home station to discover the vulnerabilities in it. They found the vulnerabilities in device firmware regarding OS command injection, stack overflow, Bluetooth stack overflow, and log file stack buffer overflow, which led to full control over the device. Likewise, the authors exposed vulnerabilities in KeyWe smart lock (https://www.iottechtrends.com/security-flaws-keywe-smart-lock-hacks/, accessed on 12 December 2021). The vulnerability found in the poorly designed communication protocol of smart lock allowed the attackers to intercept the secret pass code that is transferred between the smart lock and Android application (https://labs.f-secure.com/advisories/keywe-smart-lock-unauthorized-access-traffic-interception/, accessed on 12 December 2021). Although the KeyWe smart lock was equipped with several security mechanisms such as data encryption, the poor design of the communication protocol allowed the attackers to eavesdrop the communication between the lock and application. Unfortunately, the smart lock does not provide an option for the user to update firmware, which means that this flaw cannot be fixed. Hence, the users have to either replace the lock or keep the lock with risk.

Several vulnerabilities have been disclosed in the firmware of the Samsung SmartThings Hub (https://www.securityweek.com/samsung-patches-critical-vulnerabilities-smartthings-hub, accessed on 12 December 2021). The Samsung SmartThings Hub was designed to monitor, control, and manage the IoT devices in a smart home network. Talos Intelligence reported (https://blog.talosintelligence.com/2018/07/samsung-smartthings-vulns.html, accessed on 12 December 2021) 20 vulnerabilities in the SmartThings Hub through which an attacker can take over smart home devices. Most of the reported vulnerabilities belong to firmware or communication protocol.

Some researchers have also worked on protecting the IoT devices and networks from cyber-attacks. Feng et al. [32] proposed an automatic rules generation engine, based on vulnerability reports analysis that could be used in IDSs. However, it cannot be used to secure IoT protocols vulnerabilities due to lack of support in both the rules generation engine and existing IDSs. Similarly, in [33], the authors proposed a network filter for vulnerability protection, but it lacks defense against IoT-specific protocols exploits. Likewise, in [34], the authors proposed a solution to detect the DoS attacks in IoT 6LoWPAN networks. For this purpose, they developed IEEE 802.15.4 (layer 2) and 6LoWPAN (adaptation layer 3) protocol decoders and integrated them with an open-source intrusion detection and prevention system (IDPS). Their proposed solution firstly identifies the link type; if it matches the IEEE 802.15.4 link type, then it is decoded by their own developed decoder. Otherwise, it is decoded by the IDPS’s default layer two decoders. The authors in [35] proposed a four-layered architecture of the IDS for IoT. At first, they generated the signatures of different types of attacks by training their model on a dataset. Afterwards, they extracted the pattern of the network traffic and identified the attack if the extracted traffic pattern matches with the signatures stored in the repository.

Some researchers have also focused on MQTT protocol protection against vulnerabilities. For example, in [36], the authors developed an open source publish/subscribe system for IoT. According to the authors, MQTT is the most widely employed protocol in IoT constrained devices. In this paper, the authors presented a secure publish/subscribe system by extending MQTT using a key management framework and policy enforcement. The purpose of this system is to control the flow of information in MQTT with the help of policy enforcement. In [37], the authors presented attack scenarios and conducted security analysis of MQTT protocol. In this paper, the authors discussed various reasons why many IoT systems do not adequately implement security mechanisms. Furthermore, they discusses how MQTT protocol could be attacked by presenting various attack scenarios. In the end, they have analyzed the vulnerabilities of MQTT protocol to improve the security awareness. Additionally in [38], the authors presented an efficient fuzzy logic-based approach to detect a DoS attack in MQTT protocol for IoT called Secure MQTT. It is responsible for detecting malicious activity during the communication between IoT devices. It utilizes a fuzzy logic-based system in order to detect the malfunction behavior of the IoT nodes using a fuzzy rule interpolation mechanism.

Although the existing studies made effort to protect the vulnerabilities of IoT devices, most of them lack support for IoT-specific application layer protocols such as CoAP, MQTT, etc. Similarly, none of the above discuss works focused on protecting protocol implementation flaws. Therefore, in this study, we proposed a robust protocol parsing engine and integrated it with an existing open-source IDS in order to protect the IoT network and devices from malformed or crafted packets that may cause cyber-attacks.

## 3. Analysis of Recently Reported MQTT Protocols Vulnerabilities

In this section, we present our analysis on recently reported IoT protocol vulnerabilities that are reported in the NVD (https://nvd.nist.gov, accessed on 12 December 2021), and CVE (https://cve.mitre.org, accessed on 12 December 2021) databases. Both the NVD and CVE are famous vulnerability reporting platforms for collecting, maintaining, and disseminating detailed information regarding the latest reported vulnerabilities. As mentioned earlier, MQTT is the most popular protocol used in IoT environments. Therefore, we focused on analyzing its vulnerabilities. Particularly, in order to figure out the root causes of recently reported MQTT protocol vulnerabilities, we analyzed MQTT-related vulnerabilities reported at NVD and CVE during the 2014–2021 period.

**Figure 1:**
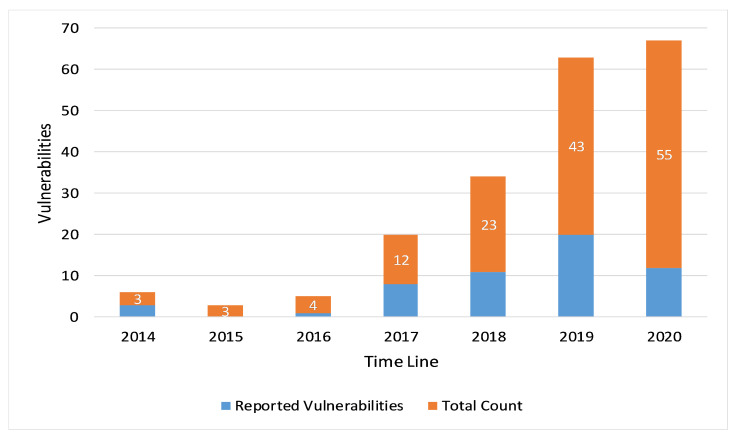
MQTT vulnerabilities reported in NVD and CVE during the 2014–2020 period.

We found that a total of 81 MQTT protocol-related vulnerabilities were reported in NVD and CVE from April 2014 to December 2021. In general, if we consider the period 2014–2020, it is possible that the number of reported vulnerabilities increase year by year (see Figure 1). The following is a description of some of the reported vulnerabilities. Upon a careful observation, we figured out that most of the MQTT-reported vulnerabilities are due to the three major issues, which include improper packet length checks, lack of required fields checks, and lack of logical errors checks. There exist some vulnerabilities that do not lie in any of these three categories: improper packet length checks, lack of required fields checks, and lack of logical errors checks; we included them in the miscellaneous category. Hence, in the overall analysis, we categorized the reported vulnerabilities into four types. The detailed analysis of each category with respect to the reported MQTT CVEs is done in the following subsections. Attackers could exploit such vulnerabilities in IoT protocols and easily craft packets with eliminated or malformed fields in order to perform different malicious activities ranging from shutting down a service to getting control over an MQTT server or IoT end device [28]. Out of 81 MQTT vulnerabilities, the number of vulnerabilities that lie in the category of improper packet length checks is 12. The number of vulnerabilities that lie in the category of lack of required fields checks is 37, while the number of vulnerabilities that lie in the lack of logical errors checks is 25.

### 3.1. Improper Packet Length Checks (LC)

**Figure 2:**
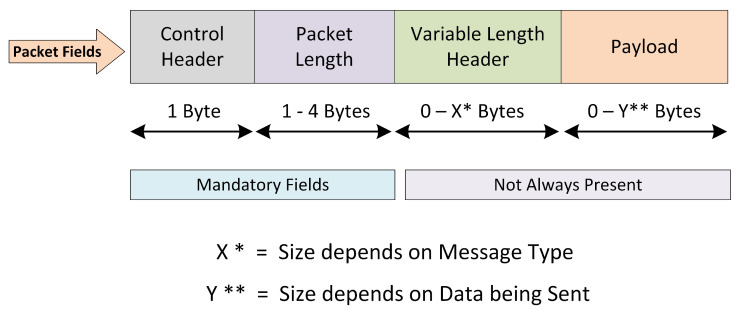
MQTT standard packet formatting.

A network packet is a formatted unit of data that is transmitted and received over the network. Figure 2 shows the formatting of a normal MQTT packet. There exist four major fields in an MQTT packet, which are control header, packet length, variable length header, and payload. The first two fields are mandatory for every MQTT packet, while the remaining ones are optional.

The extraction and identification of packet fields is called packet parsing. Although packet parsing may seem a simple activity, due to advancements in packet-crafting methods, attackers can easily exploit protocol vulnerabilities that leads to improper parsing. In particular, those that consider parsing as a sequential activity may exploit improper length checks. A large number of IoT implementation vulnerabilities are also due to missing or improper length checks before parsing. A number of improper packet length vulnerabilities of MQTT are present. We have discussed the few that follow. In CVE-2021-41036, Eclipse Paho MQTT C Client does not verify the rem_len size in readpacket. According to CVE-2020-10071, the Zephyr MQTT parsing code does insufficient checking of the length field on published messages. These situations allow a buffer overflow attack, which ultimately causes remote code execution on Zephyr. In CVE-2020-10070, the Zephyr Project MQTT code performs improper bound checking, which allows remote cute execution as well as causes memory corruption. In CVE-2019-17210, an improper check for the length and content of the MQTT topic name causes re-initialization of the main stack pointer. This situation results in DoS. Recently, a vulnerability CVE-2020-10071 reported in NVD identified the insufficient checking of packet length of published message in the Zephyr OS MQTT parsing code, which allowed the attackers to perform remote code execution and buffer overflow attack.

Some other vulnerabilities are also reported in same implementation due to improper length checks, CVE-2020-10070 and CVE-2020-10063. Similarly, another vulnerability CVE-2019-17210 was discovered in the MQTT library of Arm Mbed OS. According to the reported vulnerability, MQTT implementation suffers from improper checking while reading the topic length and content of an MQTT publish packet. Therefore, a denial of service (DoS) attack was reported when an attacker sent a crafted packet with the wrong value in topic length and content field.

In another reported vulnerability, CVE-2016-10523, the MQTT broker crashes while parsing the packet similar to <Buffer 16 03 01 01 01 01 00 00 fd …>. According to the MQTT protocol specifications, the second byte of an MQTT packet defines the remaining packet length. In the CVE-2016-10523 vulnerability, the MQTT broker crashed due to a lack of packet length validation when an attacker created a crafted packet of length 3 and tried to fill the remaining packets bytes in that buffer with more than 3 bytes. Hence, the MQTT server crashed, and a denial of service (DoS) status is reached.

The lack of packet length checks is not limited to DoS attacks, as it can also create exposure to other threats such as remote code execution (RCE) or reading memory contents. For example, in an MQTT implementation vulnerability for CVE-2017-2892, a specially crafted packet allowed the attacker to read and write the restricted memory area. Similarly, in CVE-2019-13120, due to a lack of length checks in the MQTT implementation, the attacker can read the memory contents of a device. The other similar vulnerabilities reported in MQTT implementation include CVE-2017-2894, CVE-2017-2895, CVE-2018-16528, CVE-2018-17614, CVE-2018-18764, CVE-2018-18765, CVE-2018-19417, and CVE-2018-5879. However, this issue can easily be fixed by implementing a proper length check mechanism in order to prevent the devastating attacks such as DoS, RCE, etc.

### 3.2. Lack of Required Fields Check (RFC)

The lack of required fields check is the second major cause that is found in the recently reported vulnerabilities. These vulnerabilities occur due to an ignorance of the required fields validation during the protocol implementation. As previously discussed, in MQTT, the packet length and packet fields vary with respect to the packet type. So, there should be explicit implementation of the required fields check with respect to packet type. For instance, if a packet contains a user name field, then the relative password field must also be present, as an absence of such a password section will put the implementation at a stake. We have discussed the lack of a few required fields check vulnerabilities as follows. In CVE-2019-9749, the mishandling of a crafted packet in the MQTT input plugin in Fluent Bit causes a server crash. In CVE-2018-11993, an improper check while accessing the stack upon the MQTT connection request causes a buffer overflow attack. Additionally, in CVE-2018-8531, an improper restriction of operation in the Azure IoT Hub device for MQTT protocol memory access causes a remote code execution attack. Some of the other vulnerabilities related to an improper required fields check implementation flaw are CVE-2016-9877, where the MQTT broker authenticates a connection request with a valid username but with an omitted password section, or CVE-2017-2893, where the MQTT broker crashes while processing a subscribed packet with no subscription argument.

### 3.3. Missing Logical Errors Checks (LEC)

This issue arises due to the lack of logical errors check in the packet data and lack of their identification in implementations.

We have discussed a few vulnerabilities related to a lack of logical error checks, which follow. In CVE-2021-42386, a use-after-free in Busybox’s awk applet causes DoS and possibly causes code execution when processing a crafted awk pattern. In CVE-2019-9749, the mishandling of a crafted packet in the MQTT input plugin in Fluent Bit causes a server crash. In CVE-2018-11998, a ace condition occurs while processing a crafted packet decode request in MQTT, which causes a buffer overflow attack. For instance, in CVE-2020-13849, the MQTT server implementation is vulnerable to a denial of service, and it is unable to establish new connections due to a lack of logical check on the Keep-Alive value sent by the client. The MQTT server extends a client connection timeout by 1.5 times the time asked by the client. An attacker exploited this functionality by sending a larger timeout request, keeping the server resources busy, which ultimately caused a DoS attack.

Similarly, in CVE-2019-11778, the MQTT server crashes while processing a packet having the value of “will delay interval” greater than the value of “session expiry interval”. According to the MQTT protocol specifications, the “will delay interval” should be less than or equal to the “session expiry interval”. Nevertheless, due to the missing logical errors check in the MQTT implementation, an attacker could crash the MQTT server by sending a crafted packet having the value of “will delay interval” greater than the value of “session expiry interval”.

### 3.4. Miscellaneous

While analyzing the MQTT vulnerabilities, i.e., CVEs reported at NVD and CVE, we observed some CVEs that neither belong to improper packet length checks nor belong to a lack of required fields check and missing logical errors checks. We categorized all such CVEs into the miscellaneous category. The CVEs included in this category are exploited due to an improper handling of content type, data type, authentication bypass, invalid certificates, and invalid access. The content type-related vulnerabilities reported are due to improper handling of the non-UTF-8 encoded character client ID or topic name.

For example, in CVE-2020-13932, the attacker exploited a vulnerability in Apache ActiveMQ Artemis 2.5.0 to 2.13.0 (i.e., an MQTT server) which accepts the MQTT crafted packets having non-UTF-8 encoded characters in the client ID and topic name. By exploiting such vulnerability, the attacker can execute any vulnerable script or command to obtain access over the MQTT server, hence allowing him the possibility to perform malicious activities. Similarly, the data type related vulnerabilities were reported due to an improper initialization of variables or unspecified vectors. For example, in CVE-2019-5917, a denial of service attack was performed by exploiting the unspecified vector in Microsoft Azure’s MQTT client service (i.e., azure-umqtt-c).

Instead, in case of authentication bypass-related vulnerabilities, the exploits occurred due to the unencrypted and unencoded information transformation over the network. For example, in CVE-2019-5635, due to not encrypting the data transmission between a smart bridge device and MQTT broker, an attacker exploited the MQTT broker using the default user name and password. Likewise, the invalid access-related vulnerabilities occurred due to the wrong file and objects permission. For example, in CVE-2018-8531, a vulnerability was reported that the Azure IoT Hub Device accesses objects in memory, which allowed an attacker to perform memory corruption.

## 4. Methodology

**Figure 3:**
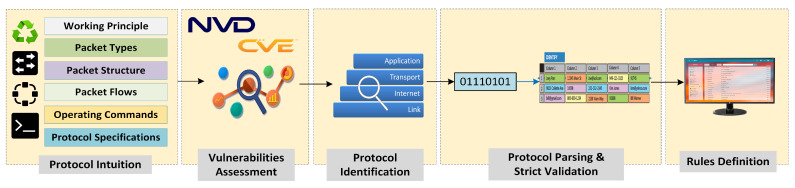
Proposed methodology for protecting the protocol-based vulnerabilities in an IoT network.

The proposed methodology for detecting and preventing the MQTT protocol implementation vulnerabilities in IoT end devices consists of five major steps, as shown in Figure 3. These steps include protocol intuition, vulnerabilities assessment, protocol identification, protocol parsing, strict protocol validations, and rules definition. In order to safeguard the IoT end devices and network from the protocol implementation vulnerabilities exploitation, the first step is the protocol intuition. In the protocol intuition step, we review information about the working principle, operating commands, packet structure, packet flows, and specifications of the MQTT protocol. After the protocol intuition, the next step is the assessment of vulnerabilities regarding the MQTT protocol from online vulnerabilities reporting platforms such as NVD and CVE. The information collected from the protocol intuition and vulnerabilities assessment is used for designing the proposed parsing engine. The proposed parsing engine is responsible for the next three major steps of the proposed methodology, which include protocol identification, protocol parsing, and packet fields validation. Finally, after the rigorous validation of incoming or outgoing packets done by the parsing engine, the further protection of the IoT network from malicious packets or attacks is performed by MQTT protocol-based rules definition. The detailed description of all the aforementioned steps is given in the following subsections:

### 4.1. Protocol Intuition

Protocol intuition is the primary and vital step of the proposed methodology for preventing MQTT protocol-based vulnerabilities. In this step, we first reviewed the working principle, key functionalities, operating commands, packet structure, packet types, packet flows, and specifications of the MQTT protocol. The MQTT is a standard, fast, light-weight, open-source, simple, and easy to implement transport protocol that is specially designed for machine-to-machine communication in an IoT environment.

**Figure 4:**
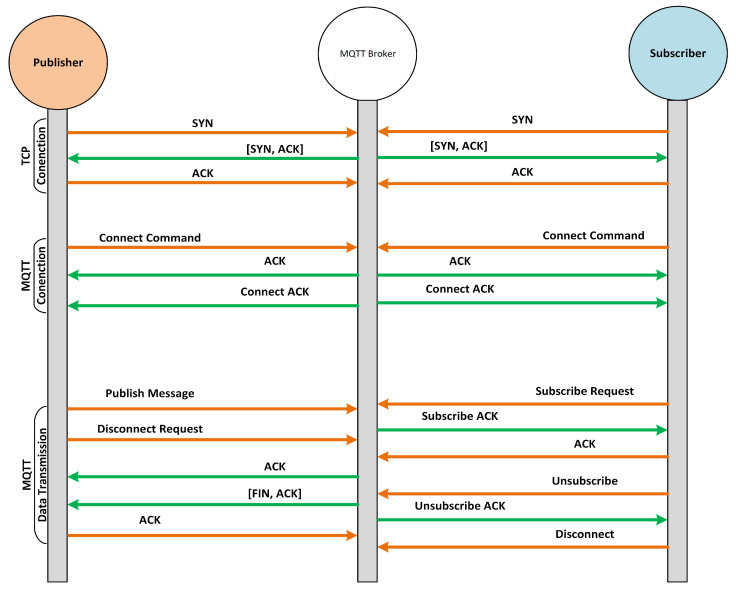
MQTT publish packet flow when QoS is set to 0.

The MQTT protocol works on the basis of the publish/subscribe model where the communication undergoes among three network entities, i.e., publisher, subscriber, and broker. The MQTT broker is basically a server through which publishers and subscribers communicate with each other. Unlike the traditional client–server model, there is no direct communication between publisher and subscriber, and all the communication between them is held by the MQTT broker. The MQTT is connection-oriented protocol where all the publishers and subscribers have to establish a connection with the broker to send or receive the messages. The MQTT subscriber becomes connected with the broker to receive the messages from the broker against a specific topic, as illustrated in Figure 4. On the other hand, the MQTT publisher becomes connected with the broker to send (publish) the messages for other clients to the broker against a specific topic, as displayed in Figure 4. Based on the publish and subscribe topics, the MQTT broker filters all incoming packets from publishers and distributes them correctly to subscribers. Both the MQTT publisher and subscriber are not aware of each other in the MQTT communication model; they are only aware of the MQTT broker.

Generally, the MQTT packet fields are classified into four major fields, which include the control header, packet length, variable length header, and payload, as previously shown in Figure 2 and described in Section 3. If we specifically consider the MQTT publish message, it generally consists of six attributes. These attributes include the packet ID, topic name, retain flag, quality of service (QoS), payload, and duplicate flag. The topic name is a simple string that may contain forward slashes for better contextual understanding. The QoS has three levels, i.e., 0, 1, 2, which determine whether the publisher needs notifications about the delivery of the publish packet to the subscribers. The retain flag defines whether the broker should save the last publish message or not. Whenever a new subscriber connects with the broker, it sends the last retained (if it exists) to the subscriber first. The payload is the actual message that a publisher sends across a topic. The message can be in the form of plain text, encrypted text, image, etc. The packet ID is for the unique identification of packets as it flows over the network. The duplicate flag is useful when QoS is set to 1 or 2. In that case, the duplicate flag is set to 1 when the publisher resends a message whose acknowledgement is not received.

**Figure 5:**
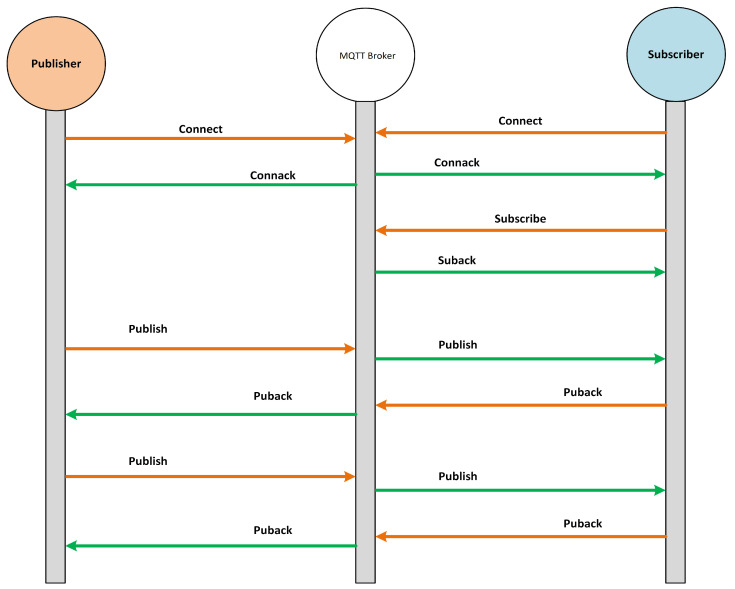
MQTT subscribe packet flow when RET flag is True and QoS is set to 2.

In case of the MQTT subscribe message, it is composed of two packet fields, i.e., packet ID and subscription list. The packet ID is for the unique identification of a packet when it goes from client to broker or broker to client. The MQTT protocol has 16 types of packets, which are released based on the QoS value and flags set in publish or subscribe packets. All these packet types along with their decimal value and the purpose for which they are released are presented in Table 1. Similarly, we overviewed the packet flows for both publish and subscribe packets. Figure 4 illustrates the packet flows when an MQTT publish client sends the publish packet with QoS 0. Likewise, Figure 5 shows the packet flows of subscribe packets when a publish packet is sent with RET 1 and QoS 2. Finally, we overviewed the MQTT protocol specifications given at (http://docs.oasis-open.org/mqtt/mqtt/v5.0/mqtt-v5.0.html, accessed on 12 December 2021) to proceed with further steps of the proposed methodology for preventing MQTT protocol-based vulnerabilities.

### 4.2. Vulnerabilities Assessment

After the protocol intuition phase is accomplished, the next step is to assess the protocol vulnerabilities in IoT devices that exist within the network. In this step, our focus is to perform an in-depth analysis of MQTT protocol vulnerabilities. For this purpose, we adopted two approaches: by surveying popular online vulnerabilities databases and by using attacking tools. For the online vulnerabilities database survey, we explored two famous online vulnerabilities reporting platforms, NVD and CVE, to investigate the online vulnerabilities reported regarding the MQTT protocol. We found a total of 81 vulnerabilities of MQTT reported from 2014 to 2021. We analyzed the major causes and the resultant devastating effects of these reported vulnerabilities. Furthermore, based on the root cause of these reported vulnerabilities, we categorized them into four types. The detailed analysis of these MQTT vulnerabilities is discussed earlier in Section 3.

Similarly, we also used two stress testing tools to send flooding traffic on port 1883 that the MQTT protocol uses for communication. These tools include MQTTSA [19] and hping3 (https://linux.die.net/man/8/hping3, accessed on 12 December 2021). We used these tools in order to check the behavior of MQTT clients (i.e., publishers and subscribers) upon receiving the flooding attack.

### 4.3. The Parsing Engine

After the protocol intuition and vulnerabilities assessment, we designed a parsing engine to detect and prevent the protocol implementation vulnerabilities. The parsing engine is the master module of the proposed methodology. It has three key functions, which include protocol identification, packet parsing, and packet fields validation. At first, the parsing engine identifies the protocol of an incoming or outgoing packet. After the protocol identification, the next step is to parse the packet with respect to the format of the identified protocol. Finally, when a packet is parsed, then it validates the packet fields. Details on such steps are reported in the following sections.

#### 4.3.1. Protocol Identification

**Figure 6:**
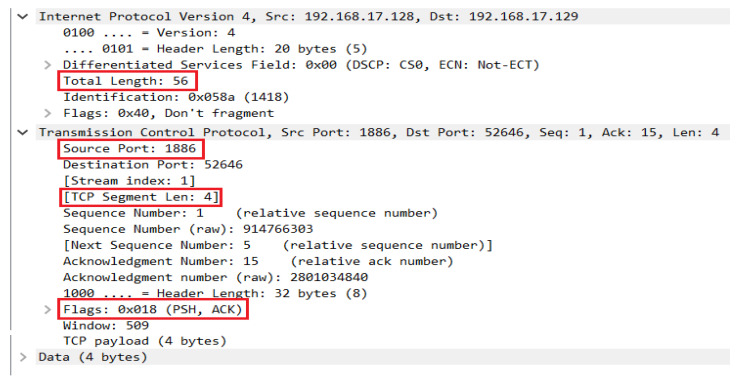
MQTT acknowledgement packet fields (highlighted in red-lined rectangle) used to identify MQTT protocol in signature-based approach.

As discussed earlier, the IoT application layer protocol-based vulnerabilities inspected during the vulnerability assessment stage can be protected using an network-based IDS. In addition, packet crafting attacks can be stopped during the packet parsing, while the flooding attacks can be protected by defining the protocol-based rules inside IDS. In order to stop IoT application layer-based packet crafting and flooding attacks, an IDS must be able to support such IoT protocols. Instead of developing an IDS from scratch, we decided to extend an existing IDS with the support of the MQTT protocol. For this purpose, we selected Suricata (version 4.1.8, released on (https://suricata-ids.org/2020/04/28/suricata-4-1-8-released/, accessed on 12 December 2021) 28 April 2020), which is a popular open-source IDS with excellent performance compared to other open-source IDSs. In order to add MQTT protocol support into Suricata, we studied its available documentation (https://suricata-ids.org/docs/, accessed on 12 December 2021) and reviewed its source code as well to understand its working principle. We found that the protocol identification in Suricata is done based on the default port number of an underlying protocol. So, at first, we performed MQTT protocol identification through a port-filtering method, i.e., detect every incoming packet at default MQTT port 1883 as an MQTT packet. Such a conventional system might get exploited if an attacker attempts a server crash attack on the MQTT broker by sending malformed packets (e.g., FTP, SMTP, HTTP, etc.) or another application layer protocol’s packets at the MQTT default port, i.e., 1883. In such a case, it is necessary to validate if the incoming packet is really an MQTT packet. Therefore, besides using the port-filtering technique, we also analyzed the sequence and features of packet flows exchanged among MQTT clients (i.e., publisher, subscriber) and the broker in order to determine the signatures of the MQTT protocol. When a publisher or subscriber wants to communicate with the broker, he needs to first get connected with the MQTT broker to send further packets on the network. Whenever an MQTT broker receives a connect request from MQTT clients, it sends a MQTT connect acknowledgement as a response. Therefore, to discover the signatures of the MQTT packet, we observed three unique characteristics in the MQTT connect acknowledgement packet. Such characteristics include the TCP length, total packet length, and flags in the response packet from the MQTT broker. We discovered that in the MQTT response acknowledgement packet, the TCP length is set to 4 bytes, the total packet length 56 bytes, and the flags value is set to 0x018 (i.e., with the Push (PSH) and Acknowledge (ACK) flag set to 1), as shown in Figure 6. So, based on these three characteristics, we determine whether the flow packets passing through the unknown ports are MQTT protocol-related packets or not.

#### 4.3.2. Packet Parsing

As previously discussed, IoT application layer protocol-based packet crafting attacks can be stopped during packet-parsing activities. Instead, flooding attacks have to be stopped by defining a protocol-based ruleset inside the IDS. A parser breaks down the incoming packets into meaningful chunks with respect to the identified protocol and packet type. Since the protocol-based rules filter network traffic across the given conditional values of keywords, therefore, a packet parser is mandatory to fire a rule for preventing an attack. In order to add MQTT protocol parsing support into Suricata, we developed an MQTT packet parser module and integrated it into Suricata source code. The packet parser checks every incoming and outgoing packet to identify whether the underlying packet is an MQTT packet or not. If the underlying packet is recognized as a MQTT packet based on the relative port number, then the packet-parsing module analyzes the MQTT packet and splits it into different fragments based on the packet type and packet length defined in the first two bytes of an MQTT packet (see Figure 2). Each MQTT packet consists of a fixed 1 byte control header in which the four most significant bits (MSBs) tell about the MQTT packet type, while the four least significant bits (LSBs) specify the flags values with respect to the packet type. The MQTT protocol has 16 types of packets. All these types are presented in Table 1 with its decimal value by which the MQTT parser recognizes the packet type.

#### 4.3.3. Strict Protocol Validations

In Suricata, we have integrated and developed a MQTT parsing engine. This engine is responsible for extensive checking against IoT protocol vulnerabilities and improper usage of MQTT protocol packet fields during the parsing stage. According to our observations, MQTT vulnerabilities are due to three major issues: improper packet length checks, lack of required fields checks, and lack of logical errors checks. These issues are due to the fact that the Suricata’s MQTT parsing engine does not perform rigorous checking against improper length checks, required fields, or logical errors. However, our developed parsing engine performs protocol validation against improper length checks, required fields, and logical errors. For example, consider the case if an MQTT packet contains a user name field; then, the relative password field must also be present, as an absence of such a password section will put the implementation at a stake. Therefore, in our developed MQTT protocol parsing engine, the engine is responsible for checking such protocol validations. The key function of our proposed parsing engine is strict protocol validations at the gateway level. It will secure devices from attackers who want to exploit protocol implementation vulnerabilities. In existing security solutions such as firewalls or IDS, the protocol implementation is only confined to the security policies or rules. For instance, an HTTP protocol parser extracts the URL from the packet to look for a specific pattern in the HTTP URL. Strict protocol implementation is different from the traditional methods. It is responsible for ensuring the guidelines provided in the protocols specification before allowing the packet to reach the target devices. Any packet that violates the protocol specifications will be stopped at the gateway level to avoid attacking the device. For instance, according to the MQTT protocol specification, only one CONNECT packet is allowed in a communication flow. Therefore, strict validation could ensure that if more than one CONNECT packet is found, the connection will be blocked. Without a strict validation, IoT devices can easily drain out their constrained resources and suffer from vulnerabilities exploitation. For development of the MQTT protocol parser, we have analyzed its specifications in detail, as mentioned in Table 1.

### 4.4. Rules Definition

As discussed earlier, several IoT devices are vulnerable to attacks due to flaws in the MQTT protocol implementation. The proposed parsing engine will protect most of the MQTT protocol implementation vulnerabilities. However, there is a need for extra security in order to safeguard the IoT network and defend against the cyber-attacks that do not belong to MQTT protocol implementation flaws, e.g., flooding attacks. For such attacks, we defined a set of rules that can be used by an intrusion detection system. The IDS will check every MQTT packet that is declared as normal by the parsing engine and block all the packets that match with the defined IDS rules. In this work, we only defined some rules for detecting and preventing the MQTT flooding attacks affecting IoT networks. For this purpose, we first reviewed the rule writing syntax of Suricata, which is based upon the protocol specific keywords whose value is extracted from an MQTT packet during the parsing stage.

So, in our case, we also defined some MQTT keywords for IDS rules writing. We further added these keywords into both the proposed parsing engine and Suricata’s rule-matching engine. The proposed parsing engine will extract the values across these keywords from every MQTT incoming or outgoing packet and perform strict protocol validations, while the Suricata’s rule engine will match the keywords’ values with the MQTT rules. If there is a rule match, then it will block that packet to safeguard the IoT network and defend against MQTT flooding attacks.

**Figure 7:**
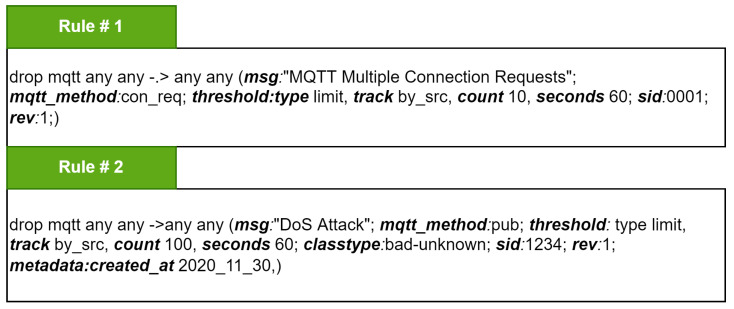
Sample rules defined in Suricata to detect and prevent MQTT flooding attacks.

Figure 7 shows two sample rules that are added into the Suricata ruleset for protecting the IoT devices and networks from flooding attacks. In the figure, rule 1 is written for detecting and stopping the multiple connection requests to MQTT broker from a single source IP address. Similarly, rule 2 detects and prevents the MQTT publish packet flood. The keywords reported in rule 1 and rule 2 are highlighted. When an MQTT packet is received by Suricata, after the strict protocol validation, the Suricata rule engine will match the values of extracted protocol keywords with its existing ruleset. When an adversary tries to send multiple connection requests to an MQTT broker, then rule 1 will be triggered, and the Suricata rule engine will drop these malicious packets if incoming connection requests from a single source exceed the limit of 10 connection requests per minute. Likewise, when an adversary attempts to flood publish packets to an MQTT broker, the Suricata rule engine will detect and drop this malicious attempt if the incoming traffic from a single source exceeds the limit of 100 publish packets per minute.

## 5. Results and Discussion

We performed a set of experiments to validate the effectiveness of the proposed solution for protecting MQTT vulnerabilities in IoT devices. We divided our evaluation into three parts. At first, we evaluated the performance of the proposed parsing engine for MQTT protocol identification over known and unknown ports traffic. Afterwards, we evaluated the performance of the proposed parsing engine for strict protocol validation. Finally, we evaluated the performance of the proposed solution for MQTT rule-based attack detection.

**Figure 8:**
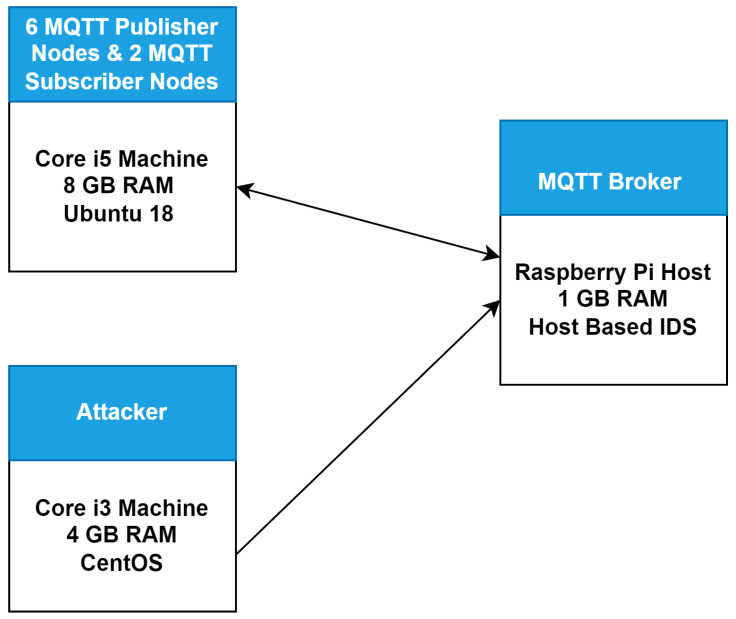
Experimental setup.

The experimental setup is represented in Figure 8. Our experimental setup consists of three machines, which include one Raspberry Pi4 host equipped with 1 GB RAM, one core i5 machine equipped with 8 GB RAM and running Ubuntu 18 OS, and one core i3 machine equipped with 4 GB RAM and running CentOS. We first installed the MQTT broker on the Raspberry Pi4 host. Then, we set up a normal network of six MQTT publisher nodes and two MQTT subscriber nodes running on the core i5 machine. The two subscribers were subscribed on the MQTT broker on two different topics. Referring to the publishers, we set up three publishers, publishing data across one topic and another three publishers publishing data across the other topic. Similarly, we set up an attack network of two attacker nodes running on CentOS machine and launching three different types of attacks against the MQTT broker. The initial experimental setup was created for testing the behavior of the MQTT broker on both normal and attack network traffic when the IDS (i.e., Suricata IDS) is not connected. We first performed all experiments without deploying the proposed solution (IDS) to observe the behavior of the MQTT broker. Afterwards, we deployed our proposed IDS into the network and then performed all the experiments to test and validate the effectiveness of the proposed solution (IDS). The following sections describe all experiments done along with their results with and without IDS.

### 5.1. Protocol Identification Testing

As previously discussed in Section 4, the proposed parsing engine first identifies the protocol of an incoming packet based on which it breaks down the incoming packets into meaningful chunks with respect to the identified protocol and packet type for protocol-based vulnerabilities prevention or rule-based attacks detection.

In order to validate the efficiency of the proposed protocol identification module, we tested it for the identification of both inbound or outbound MQTT packets traveling over the network via known or unknown ports. Thereby, we performed two experiments (in multiple attempts) with different network traffic to validate the performance of the proposed protocol identification method. While measuring the memory/CPU usage of the broker process in the following experiments, we have killed the other irrelevant processes by command. The effect of other by default running processes is negligible.

#### Experiment 1: Protocol Identification Testing over Normal MQTT and Malformed Packets

In this experiment, we first test the protocol identification module by sending normal MQTT publish and subscribe packets to the MQTT broker on its default port (i.e., 1883). For this purpose, we first connected two subscribers with the MQTT broker and subscribed them on two different topics by using the mosquitto_sub client. Afterwards, we developed a Python script to initialize six publishers, out of which three publishers started publishing messages across the first topic and three publishers started publishing messages across the other topic at a packet publish rate equal to one packet per second. The whole packets flow was passed to the protocol identification module. The protocol identification first verified the default port and then forwarded these packets to a signature-matching module. The performance comparison of results of the signature matching module over the normal MQTT packets are displayed in Table 2. Similarly, we attempted to test non-MQTT packets sent to the MQTT default port (i.e., 1883) to test the behavior of the proposed protocol identification module. For this purpose, we used the hping3 tool and sent UDP packets over destination port 1883. We also tested some publicly available non-MQTT packet capture (PCAP) files from (http://kb.boltlabs.net/knowledgebase/, accessed on 12 December 2021) to test the signature-based MQTT protocol identification. The results of the signature-based protocol identification over the malformed packets are listed in Table 2. It can be observed that the proposed protocol identification module detected MQTT packets with 100% accuracy, while it detected non-MQTT packets as non-MQTT with 100% accuracy.

### 5.2. Strict Protocol Validation Testing

In Section 3, we analyzed the MQTT protocol vulnerabilities reported on the NVD database and categorized them into four types. Later, in Section 4, we proposed a parsing engine that performs the strict protocol validation to protect the protocol implementation vulnerabilities. Thereby, in this section, we test the performance of the proposed parsing engine by performing five different experiments. The detailed description of these experiments along with results is given in the following subsections:

#### 5.2.1. Experiment 2: Testing MQTT Normal Use Case

In this experiment, we set up a normal traffic use case in which we considered six publisher nodes, two subscriber nodes, and one MQTT broker. The MQTT broker was running on a Raspberry Pi host. For running the publisher and subscriber nodes, we write a Python script for initializing and running the publisher nodes using the mosquitto-client library and a Python script for initializing and running subscriber nodes.

Afterwards, we run two MQTT subscriber nodes in order to send requests to the broker to subscribe them against two topics. Similarly, we run six publisher nodes to send publish data to the MQTT broker across two different platforms. As the MQTT broker receives a message against a topic, it forwards that message to the subscribers who have subscribed to that topic.

**Figure 9:**
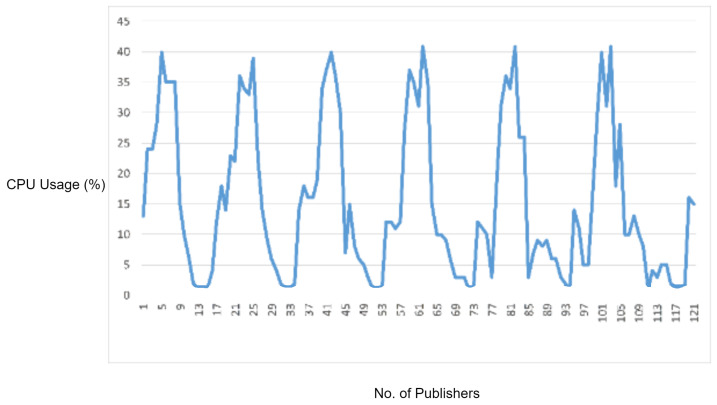
CPU usage captured while testing the normal use case.

In order to monitor the CPU and memory usage on the MQTT broker, we used the psutil Python library, providing the functionality to monitor the CPU and memory usage across a given process ID. So, we get the process ID of MQTT broker service and start monitoring the CPU and memory usage for 120 s. Figure 9 shows the CPU used by the MQTT broker during the normal traffic flow. During our tests, memory usage was measured to be almost constant, around 4–5%. Instead, the CPU usage is fluctuating during the normal traffic flow from publisher nodes to the MQTT broker and MQTT broker to the subscriber. Particularly, the CPU usage graph is fluctuating in accordance with the processing load of MQTT packets on the MQTT broker.

#### 5.2.2. Experiment 3: Testing MQTT Improper Length Check Vulnerability

**Figure 10:**
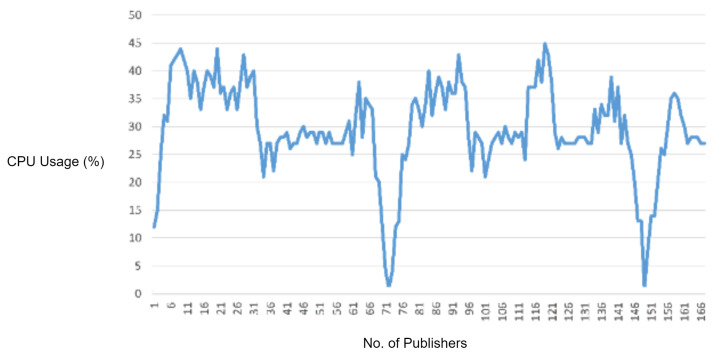
CPU usage captured while testing the improper length check vulnerability.

In this experiment, we generated a vulnerability of an MQTT packet in which we first defined a packet length and then intentionally sent fewer payload bytes than the defined packet length in order to observe the response of the MQTT broker for handling the packets with improper length. For this reason, in this scenario, we sent a malformed MQTT publish packet with the payload length less than the defined packet length. As the MQTT broker received the malware packet and it started waiting for the rest of the bytes, we flooded the malformed packet, and the overall CPU usage of MQTT broker increased, as shown in Figure 10.

In order to check the efficiency of the proposed solution, we deployed the IDS into the network and repeated the same experiment. The proposed IDS successfully detected and blocked the malformed MQTT packets. Moreover, it also reduced the CPU usage as well as legitimate traffic delay.

#### 5.2.3. Experiment 4: Testing MQTT Required Field Check Vulnerability

**Figure 11:**
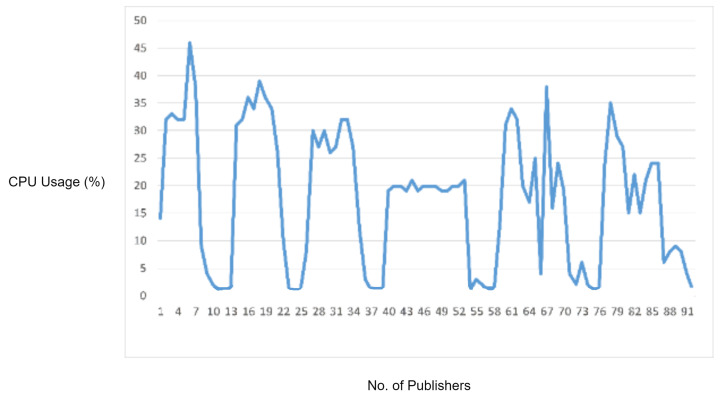
CPU usage captured while testing the required field check vulnerability.

In this experiment, we generated a vulnerability in which a required field in the MQTT packet was missing. While sending a connect request packet to the MQTT broker, the ClientID field is mandatory, and it must be the first field in the CONNECT packet payload. For generating this vulnerability, we wrote a Python script in which we skipped the ClientID field from the MQTT CONNECT packet and started flooding MQTT CONNECT packets to the MQTT broker in order to check the whether the required field check is validated or not. Although, in our MQTT version, the ClientID (required) field check was implemented, the MQTT had to process these malformed packets due to which we observed an increase in CPU usage, as shown in Figure 11. Moreover, the legitimate MQTT packets started delaying after some time. However, if these malformed packets are blocked by the IDS, then the increase in CPU utilization by the MQTT broker and delay in legitimate MQTT traffic can be prevented. Therefore, in order to check the performance of the proposed solution, we repeated this experiment with IDS deployed in the network. The proposed IDS successfully detected and blocked the malformed packets due to which there was an increase in the processing load at the MQTT broker, and the delay in MQTT legitimate traffic was prevented.

#### 5.2.4. Experiment 5: Testing MQTT Logical Error Check Vulnerability

**Figure 12:**
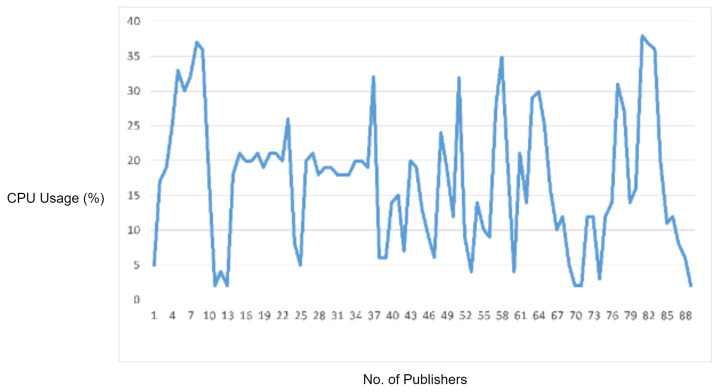
CPU usage captured while testing the logical error check vulnerability.

In this experiment, we generated a malformed packet by violating an MQTT protocol specification. In the MQTT CONNECT request, the ’Reserved’ flag bit must be zero; otherwise, the MQTT broker should disconnect the client sending such invalid request. In order to check whether the MQTT broker is validating this logical error check or not, we generated a Python script for sending a malformed MQTT CONNECT packet with the ’Reserved’ flag bit set to 1. The MQTT broker did not disconnect the client. On the other hand, when we flooded such malformed packets to the MQTT broker, the broker processed these packets to allow an invalid connection, which as a result caused an increase in CPU usage and also started delaying the legitimate MQTT traffic, as shown in Figure 12.

We also performed this experiment, after deploying the proposed IDS, the IDS easily detected and blocked all these malformed packets. Thus, the extra CPU usage of the MQTT broker and delay in legitimate traffic was averted.

#### 5.2.5. Experiment 6: Testing MQTT Miscellaneous Vulnerability

**Figure 13:**
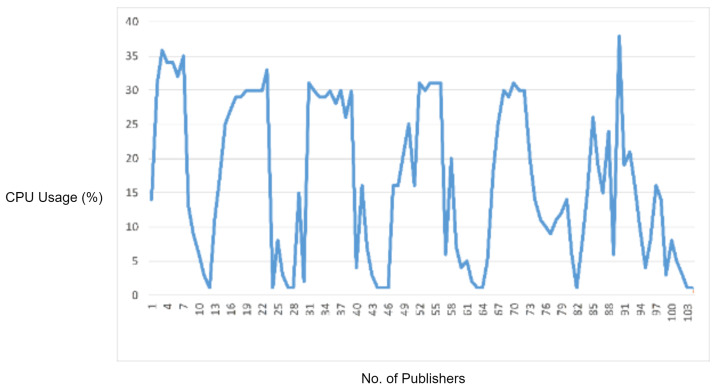
CPU usage captured while testing the miscellaneous vulnerability.

In this experiment, we generated a vulnerability from the miscellaneous category. In the MQTT packet, ClientID must be a UTF-8 encoded string, and it should only contain the characters from “0123456789abcdefghijklmnopqrstuvwxyzABCDEFGHIJKLMNOPQRSTUVWXYZ”. In order to check the MQTT broker, we sent a UTF-8 encoded string having a null character (i.e., ’\0’). By protocol specification, the MQTT broker should discard the non-UTF-8 encoded string, but in our case, MQTT accepted this packet. Figure 13 depicts the CPU utilization during the attack. Although this vulnerability did not cause a significant increase in CPU utilization, it did cause a delay in legitimate MQTT traffic. Moreover, this vulnerability can allow attackers to execute catastrophic commands that can severely harm all of the MQTT network.

In order to test the effectiveness of the proposed solution, we deployed the IDS into the network and repeated this experiment. The proposed IDS efficiently detected and blocked the malicious packet successfully; thus, it prevented the MQTT broker’s extra CPU usage as well as delay in legitimate traffic.

The experimental results show that the performance of the MQTT broker is affected when the malformed packets are sent by an attacker network. Overall, the CPU usage increased as the malformed packets were transmitted to the MQTT broker when there is no IDS deployed in the network. Moreover, the normal traffic delay also increased. In the current experiments, we considered only two attacking nodes, which also affected the performance of the MQTT broker. However, if the same attack scenario is replicated at very large scale, it can chop down the MQTT broker.

On the other hand, when the proposed IDS is deployed in the network, it detected and blocked the malicious packets from going to the MQTT broker. Table 2 validates the effectiveness of the proposed IDS. It clearly shows that the MQTT broker’s CPU usage did not increase due to malformed packets transmission when the IDS is deployed in the network. Furthermore, the normal traffic delay was also averted. Thus, the MQTT broker kept functioning normally, and all the malformed traffic is stopped at IDS.

### 5.3. Rule-Based Testing

In addition to preventing the MQTT protocol implementation vulnerabilities, we proposed rule-based MQTT attack detection for preventing the IoT devices from known attacks. As discussed earlier (in the Methodology and Introduction sections), DoS and DDoS attacks are the most common attacks in IoT environment in which the attacker first infects insecure IoT devices by installing malware on them and then instructs these infected IoT devices to perform DDoS attacks. Thus, in this section, we performed two experiments to test the performance of the proposed solution for detecting DoS and DDoS cyber-attacks through predefined signatures. The detailed description of these experiments along with results is given in the following subsections:

#### 5.3.1. Experiment 7: MQTT DoS Attack Testing

**Figure 14:**
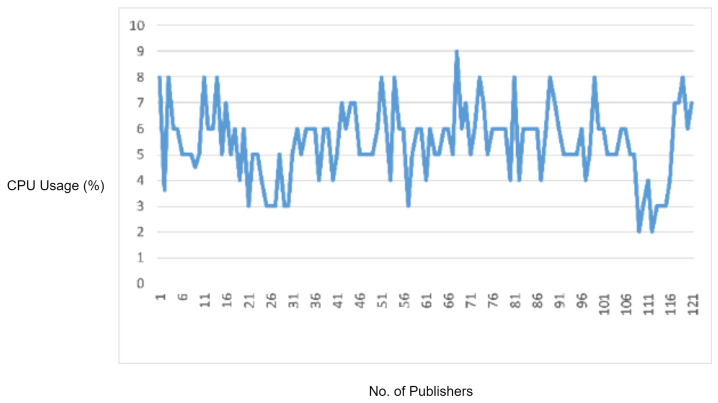
CPU usage captured while performing MQTT DoS attacks when IDS rules are disabled.

In this experiment, we developed a Python script to send multiple MQTT connection requests from a single MQTT client to the MQTT broker at a packet rate of 1000 packets per second. Figure 14 illustrates the memory and CPU usage of the MQTT broker recorded during the DoS attack when no rule (signature) was enabled in IDS.

**Figure 15:**
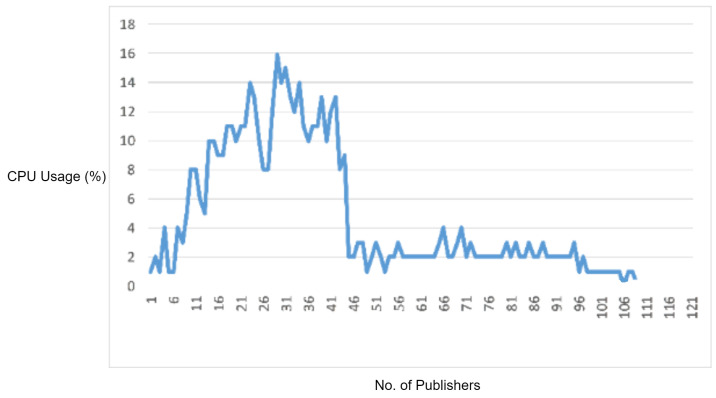
CPU Usage captured while performing MQTT DoS attack when IDS Rules are enabled.

We performed a similar attack again, but this time, we enabled attack detection signatures developed to detect and stop the DoS attack. Figure 15 shows that at the start, the CPU and memory usage of the MQTT broker was high, but as the rule-based engine detected the DoS attack based on the signature matching, it stopped further communication from the attacking device. Hence, the CPU and memory usage became normalized as the rule-based engine detected and stopped the DoS attack packets coming toward the MQTT broker.

#### 5.3.2. Experiment 8: MQTT DDoS Attack Testing

In order to test the effectiveness of the rule-based engine for detecting and stopping the DDoS attacks, we used an MQTT DDoS attacking tool, i.e., MQTTSA [19]. The MQTTSA [19] tool is capable of generating MQTT protocol-based DDoS attack by sending the MQTT connection requests, and MQTT publishes messages through port switching, i.e., sending flooding packets through multiple source ports of an underlying attacking device to disturb the normal working of the target device (i.e., the MQTT broker).

**Figure 16:**
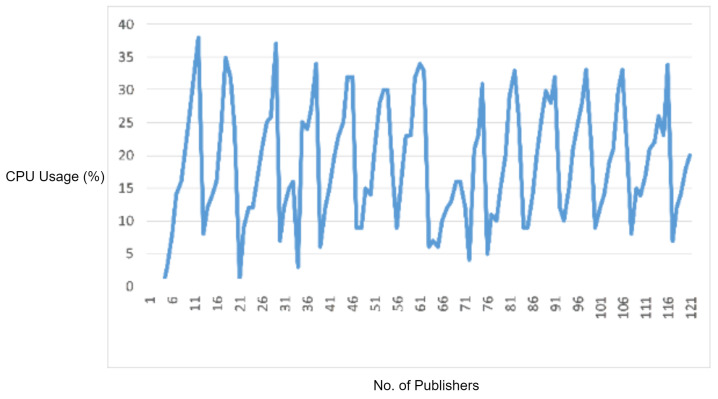
CPU usage captured while performing MQTT DDoS attack when IDS rules are disabled.

In this experiment, we sent multiple MQTT connection requests and MQTT publish packets at a packet rate of 3000 packets per second to the MQTT broker. We first performed the DDoS attack using the MQTT-SA [19] tool without enabling the rule engine of IDS and monitored the CPU and memory consumption by the MQTT broker. The obtained observations are visualized in Figure 16.

**Figure 17:**
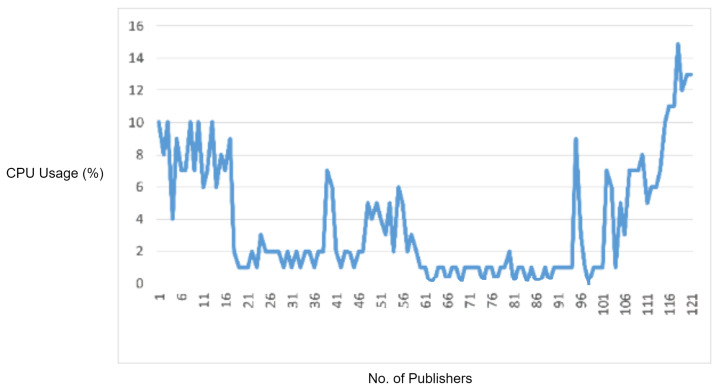
CPU usage captured while performing MQTT DDoS attack when IDS rules are enabled.

In the next attempt, we enabled IDS rules and performed a similar DDoS attack to test whether the DDoS attack is detected or not. According to our DDoS detection signatures, the rule engine detected the DDoS attack as a flow—a flow is a combination of five tuples, i.e., source IP, source port, destination IP, destination port, and protocol—that crosses the limit of sending an MQTT connection request or MQTT publish packets. In our scenario, we set the packet rate limit up to 500 packets per second. As an attacking flow crosses this limit, the DDoS detection rule is fired, and that flow is blocked by IDS for further communication. Figure 17 displays the CPU and memory usage of the MQTT broker during the DDoS attack when the IDS signatures were enabled. It can be observed that at the start, the CPU and memory usage increased, but after some time, the attacking flow crossed the defined packet limit, i.e., 500 packets per second, and the DDoS attack detection signature detected and blocked the attacking flow. Thus, the CPU and memory usage of the MQTT broker came back to its normal condition as the attack was stopped by the IDS rule engine.

In the following, we report a brief summary of the obtained results. Table 3 also provides a brief overview of the results and experimentation. In this, we have listed the experiments performed and have summarized their results. The detected column shows that the particular experiment has successfully detected the packet of MQTT under consideration. Similarly, the blocked column in the table indicates that the particular experiment has successfully blocked the malformed packet under consideration. Meanwhile, the CPU usage column shows the CPU usage during the detection of malformed or normal MQTT packets.

In the experimental results figures above (Figure 9, Figure 10, Figure 11, Figure 12, Figure 13, Figure 14, Figure 15, Figure 16 and Figure 17), the CPU usage is varying in accordance with the load on the CPU (MQTT broker). When the load is high, the value of the usage is high and vice versa. Here, the CPU usage of the MQTT broker varies in accordance with the processing load of the MQTT packets on it. Much processing power is required by the CPU when packets are malformed. When the load increases, CPU usage increases, and when the load decreases, CPU usage decreases.

Particularly, in order to validate the effectiveness of the proposed solution for protecting MQTT vulnerabilities in IoT devices, we have performed a set of experiments as mentioned above. By doing this, we have evaluated the performance of the proposed parsing protocol engine.

Firstly, we have evaluated the protocol identification module of the protocol parsing engine, in which we have done protocol identification testing over normal MQTT and malformed MQTT packets. According to the results, the proposed protocol identification module detected MQTT packets with 100% accuracy.

Next, we have evaluated the performance of the protocol parsing engine by performing different experiments. In the first experiment, we have evaluated the protocol parsing engine by testing it with an MQTT normal traffic use case. The experiment shows the CPU and memory consumption used by the MQTT broker during the normal traffic flow. According to the experimental results, the memory usage is constant, while CPU usage values are fluctuating in accordance with the workload of MQTT packets on the MQTT broker CPU.

Then, the protocol parser engine is tested over MQTT improper length vulnerability. According to the results, the overall CPU usage of the MQTT broker increased because of extra CPU processing caused by malformed MQTT improper length packets. The protocol parser engine detected these improper length packets with 100% accuracy.

Hence, the protocol parser engine is tested over MQTT malformed packets in which a required field in the MQTT packet was missing. According to the results, an increase in the CPU usage of the MQTT broker has been observed due to the extra processing caused by malformed packets. The proposed protocol parser engine attached with IDS successfully detected and blocked the malformed packets. In virtue of this, the increased processing load on the MQTT broker and delay in MQTT legitimate traffic has been prevented.

As an additional experiment, the protocol parser engine has been tested over MQTT malformed packets which have been crafted by violating an MQTT protocol specification. According to the results, the CPU usage of the MQTT broker has been increased due to these malformed packets as the broker processed these packets to allow invalid connection, which as a result caused an increase in CPU usage and also a delay of legitimate packets processing. We measured that the proposed protocol parser engine, connected with the IDS, successfully detected and blocked malformed packets, and therefore, extra CPU usage has been prevented.

Furthermore, as another experiment, we tested miscellaneous MQTT vulnerabilities exploited over the proposed protocol parser engine. According to the results, the vulnerabilities do not cause any significant increase in the CPU usage of the MQTT broker. However, they caused delays in legitimate MQTT traffic processing. The proposed protocol parser engine successfully detected and blocked such vulnerabilities attacks.

Additionally, we have executed rule-based testing of the proposed protocol parser engine by MQTT DoS attack testing and MQTT DDoS attack testing. For both attacks, the CPU usage and memory usage of the MQTT broker came back to its normal condition as the attack is stopped by the protocol parser-enabled MQTT IDS rule engine.

## 6. Conclusions and Future Work

In this paper, we analyzed the recently reported vulnerabilities of MQTT, a popular IoT application layer protocol. In our analysis, we categorized the reported vulnerabilities into four types.

Based on the four major causes reported in these vulnerabilities, we proposed a methodology to protect the IoT network and end devices from these vulnerabilities. The proposed solution consists of a parsing engine, which is integrated with an existing open-source IDS, Suricata. The proposed parsing engine analyzes every incoming and outgoing packet based upon the strict protocol specifications validation. If an invalid/malformed packet is detected, then it blocks that packet, thus protecting the IoT devices and network vulnerabilities.

In order to test the effectiveness of the proposed solution, we performed a set of experiments by generating both the normal and malformed traffic. The experimental results prove how the proposed solution efficiently detects all the malformed packets and blocks them at the network level before they can reach and exploit IoT devices.

The existing work is limited to the MQTT protocol. Therefore, in the future, a similar analysis could be done for other IoT application layer protocols to analyze the major causes of the vulnerabilities reported in those protocols. The second limitation of this work is that the proposed parsing engine cannot handle every reported vulnerability of MQTT. Currently, it can handle the vulnerabilities that violate MQTT protocol specifications by which many of the recently reported and zero-day vulnerability exploitation attacks can be protected, even if the underlying IoT devices connected in a network do not have the ability to perform strict protocol validations. In the future, we aim to expand this work to other IoT protocols and propose a generic parsing engine that can perform strict protocol validations for every IoT application layer protocol.

## Figures and Tables

**Table 1:** MQTT packet types and their values defined in four MSBs.

Packet Type	Description	Value
Reserved	Reserved	0
CONNECT	Connection request	1
CONNACK	Connection acknowledgment	2
PUBLISH	Publish message	3
PUBACK	Publish acknowledgment	4
PUBREC	Publish received	5
PUBREL	Publish released	6
PUBCOMP	Publish complete	7
SUBSCRIBE	Subscribe request	8
SUBACK	Subscribe acknowledgment	9
UNSUBSCRIBE	Unsubscribe request	10
UNSUBACK	Unsubscribe acknowledgment	11
PINGREQ	Ping request	12
PINGRESP	Ping response	13
DISCONNECT	Disconnect notification	14
Reserved	Reserved	15

**Table 2:** CPU and memory utilization by MQTT broker in five experiments when performed with and without the proposed IDS.

	without IDS		with IDS	
Use Case	Avg. CPU (%)	Avg. Memory (%)	Avg. CPU (%)	Avg. Memory (%)
Normal	15.15	0.54	15.15	0.54
RFC	17.70	0.54	15.15	0.54
LEC	17.21	0.54	15.15	0.54
LC	29.59	0.54	15.15	0.54
Misc.	16.78	0.54	15.15	0.54

**Table 3:** Experimentation and results summary.

No.	Experiment	Detected	Blocked	CPU Usage
1	Protocol Identification Testing Over Normal MQTT and Malformed Packets	Yes	-	Fluctuating as normal, High with malformed
2	Testing MQTT Normal Use Case	Yes	-	Fluctuating
3	Testing MQTT Improper Length Check Vulnerability	Yes	Yes	High
4	Testing MQTT Required Field Check Vulnerability	Yes	Yes	High
5	Testing MQTT Logical Error Check Vulnerability	Yes	Yes	High
6	Testing MQTT Miscellaneous Vulnerability	Yes	Yes	High
7	MQTT DoS Attack Testing	Yes	Yes	High
